# Minimum Appropriate Dose of Lidocaine with a Fixed Dose of Sufentanil Epinephrine Used for Spinal Anesthesia in Caesarian Section

**DOI:** 10.5812/aapm.7810

**Published:** 2013-01-01

**Authors:** Parisa Golfam, Mitra Yari, Hamid Reza Bakhtiyari

**Affiliations:** 1 Department of Anesthesiology, Kermanshah University of Medical Sciences, Kermanshah, Iran; 2 Clinical Research Development Center, Kermanshah University of Medical Sciences, Kermanshah, Iran

**Keywords:** Cesarean Section, Lidocaine, Anesthesia, Spinal, Sufentanil

## Abstract

**Background:**

Caesarian section is a commonplace surgery in females for which spinal anesthesia is the preferred method. The local anesthetic medications used in the surgery are often associated with complications such as nausea, vomiting, dyspnea, hypotension, and bradycardia. In the present study, we decreased the dose of the anesthetic drug and added an opioid instead.

**Objectives:**

We tried to find an appropriate combination of medications required for optimal anesthesia with minimum complications.

**Patients and Methods:**

One hundred twenty six candidates for C/S with first and second class ASA aged 18-35 years were randomly divided into three groups. All patients received sufentanil (2.5 µg) and epinephrine (100 µg) doses but the lidocaine doses were respectively 50 mg, 60 mg and 75 mg in the groups 1, 2 and 3. Complications including hypotension, bradycardia, dyspnea, nausea, vomiting, and anesthesia quality were recorded and statistically analyzed.

**Results:**

The level of anesthesia was significantly different between groups. By reducing the lidocaine dose, patients with anesthesia level under the nipple increased but the surgeon and the patient were satisfied with the results. Nausea, vomiting, and dyspnea was degraded by decreasing the lidocaine dose especially in the 50 mg group. The need to use ephedrine was directly associated with the lidocaine. However, the need to use atropine was not significantly different between groups. Pruritus was not significantly different as well.

**Conclusions:**

It seems that reducing the lidocaine dose, when combined with sufentanil, decreases most complications of spinal anesthesia such as hypotension, dyspnea, nausea, and vomiting while preserving anesthesia quality.

## 1. Background

Cesarean section (C/S) is among the most common surgeries in females with spinal anesthesia being the most frequent anesthetic method. Local anesthetic drugs such as bupivacaine and lidocaine are usually used in spinal anesthesia. Lidocaine 5% is among the most common drugs used in most medical centers in Iran. Spinal anesthesia with local anesthetic drugs cause the desirable effects of a sensory and motor block, but may also cause undesirable effects such as complications like hypotension, bradycardia, dyspnea, nausea and vomiting (1). In order to have a better quality of spinal anesthesia, and a longer duration of analgesia following surgery, opioid drugs such as sufentanil can be added to lidocaine (2, 3). Former researches on the subject were primarily focused on an increase in analgesia duration and improved block quality (2, 4). In some of the studies, the effects of adding an opioid in the context of complications of spinal anesthesia have been evaluated (5). Some authors have assessed the effects of the added opioid on fetus general health. The dose of the drugs used and sometimes the type of the local anesthetic or opioid drugs has been different in these studies. In the present study, we used a fixed dose of sufentanil in combination with different doses of lidocaine in order to minimize its complications. Our goal was to determine the minimal dose of lidocaine needed to achieve an appropriate level of anesthesia and evaluate the quality of the analgesia.

## 2. Objective

In this investigation, we tried to determine the minimum dose of lidocaine required for spinal anesthesia in addition to a fixed dose of sufentanil and epinephrine.

## 3. Patients and Methods

Following obtaining ethical approval from Kermanshah University of Medical Sciences, 126 pregnant women aged 18-35 years with height of 150-170 centimeters and a gestational age of 36-40 weeks who were in the first and second class of ASA were included in the study following an informed written consent. Women with an underlying disease, those suffering from a chronic pain, patients who have used analgesic drugs during the past two days, and those with any contraindications for spinal anesthesia were excluded. The patients were selected by convenience sampling and then randomized into three equal groups (42 subjects in each group calculated with 80% statistical power and 95% confidence level). Spinal anesthesia was induced by a combination of epinephrine, Lidocaine and sufentanil. In all of the three groups, the sufentanil dose (2.5 µg) and epinephrine dose (100 µg) were fixed; however, the lidocaine dose was different in the three groups (50 mg in group 1, 60 mg in group 2 and 75 mg in group 3). In all of the groups, 10 cc/kg of ringer serum was infused prior to inducing anesthesia. Spinal anesthesia was induced by a Quincke needle (25G) in the lumbar intervertebral space of L3-L4 or L4-L5 while the patient was in a sitting position; then, the patients were immediately laid on the bed and the bed was placed in a flat position. Spinal anesthesia was conducted by an anesthesiologist who was not involved in the ensuing monitoring, evaluation, and data recording. Furthermore, those completing the checklists and evaluating the symptoms were not aware of the patients’ status within each of the groups. The patients’ blood pressure and heart rate were recorded every three minutes by a Datascope monitoring system. If the blood pressure fell to 25% of the basic level or the systolic blood pressure decreased to less than 95 mmHg, the patient received an intravenous bullous of 5-10 mg ephedrine. Similarly, if the heart rate was less than 50, 0.5 mg atropine was administered. All of the patients received an oxygen mask during surgery and if the arterial oxygen saturation dropped below 92%, which is defined as respiratory depression, it was recorded. Complications such as nausea, vomiting, dyspnea and pruritus were also recorded. Anesthesia level was assessed by a needle in the midline and the nipple was the basis. It was defined as upper as nipple, at the level of nipple and lower than nipple. The collected data were statistically analyzed. For comparing the qualitative variables such as nausea, etc., the x^2^ test was used. For the ordinal variables, the x^2^ test and Spearman correlation coefficient were applied. ANOVA and Tukey post hoc tests were used for the quantitative variables such as analgesia duration after surgery. To match the groups, the same tests or stratification method was used based on the variables. SPSS version 12 was used for analysis and *P* value < 0.05 was considered as significant.

## 4. Results

The demographic data were not significantly different between the three groups. The level of analgesia was significantly different between the studied groups (*P* < 0.001). By reducing lidocaine dosage (group 1), the number of the patients with analgesia level lower than nipple increased (Table 1); however, neither the surgeon nor the patients were dissatisfied. Four patients in 50 groups, five in 60 groups, and four in 75 groups received Ketamine (20 mg) to supplement analgesia. This finding was not statistically significant. Nausea, vomiting and dyspnea was significantly different between the groups and these symptoms were reduced by the decreased lidocaine dose especially as seen in the 50 mg group. The necessity to use ephedrine showed a significant difference between the groups and was directly associated with decreasing the dosage of lidocaine. With respect to use of atropine, there was no significant difference between the three groups. Pruritus was not significantly different between the evaluated groups (Table 2).

**Table 1.** Frequency of the Analgesia Level in Various Groups

Analgesia Level Drug Dose	Upper nipple, No.	Nipple, No.	Under nipple, No.
**75 mg group**	19	12	11
**60 mg group**	9	8	25
**50 mg group**	7	3	32

**Table 2.** Comparison of Side Effects between Groups

	Group 1 (50 mg), %	Group 2 (60 mg), %	Group 3 (75 mg), %	*P* value
**hypotension**	26%	76%	73%	< 0.001
**vomiting**	0.07%	11%	33%	< 0.001
**nausea**	16%	26%	45%	< 0.001
**dyspnea**	0.02	19%	28%	0.004
**bradycardia**	o.02	0.09	0.09	0.342
**pruritus**	0.02	0.02	0.04	1.000

## 5. Discussion

Our investigation suggests that reducing the dose of lidocaine decreases complications of spinal anesthesia including nausea, vomiting, hypotension and dyspnea and in order to compensate and preserve the quality of analgesia, adding 2.5 µg of sufentanil (0.5 cc) is sufficient as well. Furthermore, when a low dose of opioid is used, its side effects are reduced. For example, none of the patients in this study had respiratory depression and the incidence of pruritus was also very low. Despite reducing the level of analgesia in Group 1, the patients underwent the surgery without pain and the surgeon did not express any dissatisfaction as well. This fact demonstrates that reducing the block level does not compromise block quality, and also provided more stable conditions for the patients because of a less sympathetic blockade. The data indicates that reducing dose of the local anesthetic drug decreases many complications such as nausea, vomiting, dyspnea and severe hemodynamic changes that need medical interventions by providing proper surgical conditions for patient and surgeon. The need for atropine was not directly associated with the need for ephedrine (which was significantly different) which might be due to the compensatory tachycardia in response to the patients’ hypotension or influenced by the tachycardia resulted from ephedrine used in the treatment of the hypotension. Respiratory depression was not observed in any of the patients. In most of the studies using Sufentanil in combination with a local anesthetic, high doses of opioid in the range of two to four times of that used in this study has been applied (4, 5-7). In some of the studies, fentanyl has been used instead of Sufentanil (2, 3, 8) and some others have compared these two opioids in such a combination. For example, Nelson et al. when comparing these two opioids concluded that Sufentanil is a better option with a potency of 4.5 times of fentanyl (9, 10). In contrast with the present study, in most of the studies evaluating the combination of opioid and local anesthetic in spinal anesthesia, different doses of opioids with a fixed dose of local anesthetic drug have been used. As a result, no reduction in the complications of spinal anesthesia has been observed (4, 5, 8, 9, 11). In some studies like ours, different doses of bupivacaine was used in combination with Sufentanil or Fentanyl and the results were consistent with the present study; however, because of using higher dose of Sufentanil in some studies pruritus was higher than the present study (12, 13). Some other researchers have only evaluated the effect of opioids on the quality and duration of the block without considering its effect on the complications of spinal anesthesia (3). In a study by Bakhshaei et al. using high dosage of lidocaine (75 mg) in combination with high dosage of Sufentanil, although analgesia duration and quality was high, there was no difference in nausea, vomiting and hypotension reported and the complications limited to opioid-like respiratory depression have been further assessed (4). Bayat et al. using low-dose lidocaine in combination with moderate-dose Sufentanil 5 µg (1 cc) have concluded that the complications of spinal anesthesia in the both groups were the same and pruritus in the group receiving Sufentanil was about 22% (6). In a study by Palmar et al., 80 mg of lidocaine with normal saline has been injected in one group while the other group received the same dose of lidocaine with 15 µg of Sufentanil. The duration of analgesia in the group receiving the opioid with the local anesthetic was higher while nausea and vomiting was lower. However, the incidence of pruritus was not different between the two groups (2) which is not consistent with our study because in this study dosage of lidocaine has not been reduced which resulted in no reduction in the incidence of nausea and vomiting. Demriran et al. used a low dosage of sufentanil in combination with bupivacaine and concluded that low doses of sufentanil of about 1.5 mg and 2.5 mg significantly decreased incidence of pruritus in addition to providing an appropriate analgesia for C/S. These authors have suggested low doses of sufentanil (12). Although in most of the studies focusing on the effect of intrathecal opioid on fetus health, no fetal complication has been reported (5, 10, 14-16). There are reports about the adverse effect of intrathecal opioid in fetus heart rate in doses higher than our study (17-19). It has been suggested that lower dose of opioid are used as in the present study; however, there is controversy in this issue (adverse effect on fetus heart rate). The complication of pruritus due to opioids, which is sometimes very harrowing, is directly associated with opioid dosage as well (20, 21). Using lidocaine for spinal analgesia has been less frequent because of transient neurologic syndrome and has been replaced by bupivacaine in most centers (22). However, we used this medication for the reason that it is the most commonly used drug in our center. Besides, transient neurologic syndrome was observed in none of our patients. Since the prevalence of this syndrome is directly associated with the increased dosage of lidocaine, using a minimum dose of lidocaine in combination with sufentanil instead of using only a high dose of lidocaine can be done in order to reduce this syndrome and is recommended for the centers with a limited access to bupivacaine. According to the findings of our study, it can be indicated that using lidocaine in dose of 50 mg, instead of 75 mg, in combination with opioids and epinephrine in candidates for cesarean section not only provides an acceptable anesthesia level but also offers a more stable hemodynamic state with fewer side effects and less need for drugs for to control blood pressure and vomiting.
